# Swearing enhances manual dexterity

**DOI:** 10.3389/fpsyg.2025.1676618

**Published:** 2026-01-07

**Authors:** Nicholas B. Washmuth, Dominic Bromley, Kieran Bromley, Christopher G. Ballmann, Sophia L. Porrill, Richard Stephens

**Affiliations:** 1 Department of Psychology, The University of Alabama in Huntsville, Huntsville, AL, United States; 2 Department of Physical Therapy, Samford University, Birmingham, AL, United States; 3 School of Psychology, Keele University, Keele, United Kingdom; 4 School of Medicine, Keele University, Keele, United Kingdom; 5 Department of Human Studies, University of Alabama at Birmingham, Birmingham, AL, United States; 6 School of Health Profession, UAB Research Collaborative, University of Alabama at Birmingham, Birmingham, AL, United States

**Keywords:** manual dexterity, Minnesota Manual Dexterity Test, Nine-Hole Peg Test, psychological warm-up, swearing

## Abstract

Swearing, or the use of taboo language, has been linked to increased psychological flow, self-confidence, and state disinhibition, raising the possibility that it may function as a psychological warm-up to enhance fine motor skills. This study investigated whether repeating a swear word, compared to a neutral word, improves manual dexterity. Two randomized, repeated-measures experiments were conducted. Experiment #1 (*n* = 61) assessed manual dexterity using the Minnesota Manual Dexterity Test (MMDT) and state disinhibition via the Stop-Signal Reaction Test (SSRT). Experiment #2 (*n* = 42) used the Nine-Hole Peg Test (9-HPT) and examined the moderating role of daily swearing frequency. In both experiments, participants repeated a self-selected swear or neutral word for 15 s before performing the dexterity task. Swearing significantly improved performance on both the MMDT (*p* < 0.001, η*^2^_*p*_* = 0.268) and the 9-HPT (*p* = 0.036, η*^2^_*p*_* = 0.105). However, no differences in state disinhibition were observed between conditions, and daily swearing frequency did not moderate the effects. These findings suggest swearing enhances manual dexterity, but mediating and moderating factors remains unclear.

## Introduction

Psychological warm-ups prepare individuals for high-performance tasks by optimizing mental and physiological states. Elite athletes consistently engage in psychological warm-ups during both training and competition, distinguishing themselves from recreational and novice athletes (Arvinen-Barrow et al., 2007). Research identifies psychological warm-up strategies as a hallmark of Olympic-level competitors (Taylor et al., 2008). Beyond elite sports, psychological warm-up strategies have also been shown to enhance performance on tasks requiring fine motor control (Perry and Katz, 2015).

The goal of psychological warm-up strategies is to create an optimal state for peak performance. These strategies enhance arousal, attentional control, and self-confidence (Orlick, 2016). Arousal, defined as an elevated state of physiological and psychological energy, plays a crucial role in task execution (Tynes and McFatter, 1987; VaezMousavi et al., 2009). Attentional control, the ability to focus on a task while inhibiting distractions, is critical in high-pressure situations such as emergency response and driving (Marquardt et al., 2018, 2023). Similarly, self-confidence, the belief in one’s ability to succeed in a given situation, consistently predicts success across various high-stakes environments (Brewer et al., 2019; Perry, 2011).

Swearing, defined as the use of taboo words with the potential to offend (Beers Fägersten, 2012), represents a unique psychological behavior with potential relevance to performance. Research suggests swearing can provide psychological benefits (Stapleton et al., 2022), including increased self-confidence, enhanced psychological flow (a state of optimal engagement), increased state disinhibition (a reduction in restraint) (Stephens et al., 2022), and improved mood (Ballmann et al., 2024). Swearing has also been shown to improve pain tolerance (Hay et al., 2024; Stephens et al., 2009; Stephens and Umland, 2011; Stephens and Robertson, 2020) and enhance performance on short, intense physical tasks (Washmuth et al., 2024). However, the effects of swearing appear susceptible to habituation, where repeated exposure reduces psychological responses. For example, habituation to swearing diminishes its hypoalgesic effects (Stephens and Umland, 2011). These findings suggest that any potential performance benefits of swearing may decrease with habitual swearing.

Given swearing’s association with psychological states conducive to high-demand environments, such as elevated engagement, self-confidence, and reduced inhibition, it is plausible that swearing may function as a psychological warm-up. This raises an intriguing question: Could swearing enhance performance on tasks requiring fine motor control and precision? Manual dexterity, the ability to skillfully manipulate objects using coordinated hand and finger movements, is essential in domains such as surgery, keyboarding, sewing, and playing a musical instrument. Interestingly, surgeons, who exhibit high levels of manual dexterity, have been observed to swear during surgical procedures and other fine motor tasks (Joseph et al., 2024; Palazzo and Warner, 1999). Furthermore, research indicates that psychological preparation improves performance on dexterity-based surgical tasks (Kahol et al., 2009). Thus, swearing may serve as a practical psychological warm-up strategy for tasks requiring manual precision, accuracy, and attentional focus. Thus, the purpose of this study is to examine whether swearing can improve manual dexterity by serving as a psychological warm-up. Two experiments were conducted to investigate this theory. Given the exploratory nature of these unregistered studies and the lack of prior research, findings should be viewed as preliminary evidence of swearing’s potential as a psychological warm-up for fine motor tasks.

## Experiment 1

The first experiment aimed to determine whether swearing impacts manual dexterity and whether state disinhibition mediates this effect. It was hypothesized that (1) repeating a swear word prior to the manual dexterity task would improve performance, resulting in faster manual dexterity scores in the swearing condition compared to the neutral word condition, and (2) that swearing would lead to increased state disinhibition.

### Methods

#### Participants

Sixty-one participants (42 females; *M* = 21.61 years, SD = 4.26 years) were recruited from a convenience sample of Keele University students. Exclusion criteria included individuals with any health condition affecting manual dexterity. Informed consent was obtained prior to data collection, and this study was approved by the Keele University Psychology Ethics Committee.

#### Materials

##### Minnesota Manual Dexterity Test (MMDT)

**FIGURE 1:**
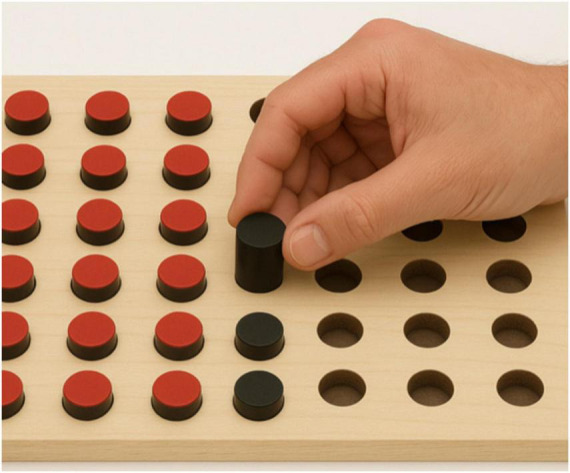
Minnesota Manual Dexterity Test. Figure created by ChatGPT-4.

The MMDT is a standardized test of manual dexterity, involving four different tasks, with this experiment using the one hand turn and flip task (Lourenção et al., 2005; Figure 1). The MMDT has been shown to be a reliable and valid measure of manual dexterity (Desrosiers et al., 1997). Participants were required to use their dominant hand to pick up each peg, one at a time, turn it over in the hand, and place it back into its original hole, as quickly as possible (Duncan et al., 2015). The time taken to complete the task, measured in seconds, was recorded.

##### Stop-Signal Reaction Time test

Participants completed the Stop-Signal Reaction Time (SSRT) test using the pre-coded Stop-It program (Verbruggen et al., 2008) to assess response inhibition, the ability to stop a planned motor response. Participants responded to a “go” signal by pressing left or right arrow keys, with some trials including an auditory “stop” signal instructing them to withhold their response. The stop signal delay (SSD) dynamically adjusted based on performance to maintain a challenging level of inhibition. Reaction time and success rates were recorded, with longer reaction times and lower accuracy indicating greater state disinhibition.

##### Vocalization

Participants self-selected their swear and neutral words in response to two prompts: what word would they say if they hit their thumb with a hammer (swear word), and what word they would use to describe a table (neutral word).

#### Design and procedure

A repeated measures design was utilized, with the order of condition (swear word, neutral word) randomized and counterbalanced. The dependent variables were the time required to complete the MMDT, reaction times on the SSRT, and success rates on the SSRT.

Depending on the randomized condition order, participants were instructed to repeat their chosen swear word or neutral word for 15 s, at normal speech volume and maintaining a steady pace of once every second, before completing the MMDT and SSRT. They were silent during task completion. A 5-min washout period between conditions was used to minimize potential carryover effects.

### Results

**FIGURE 2:**
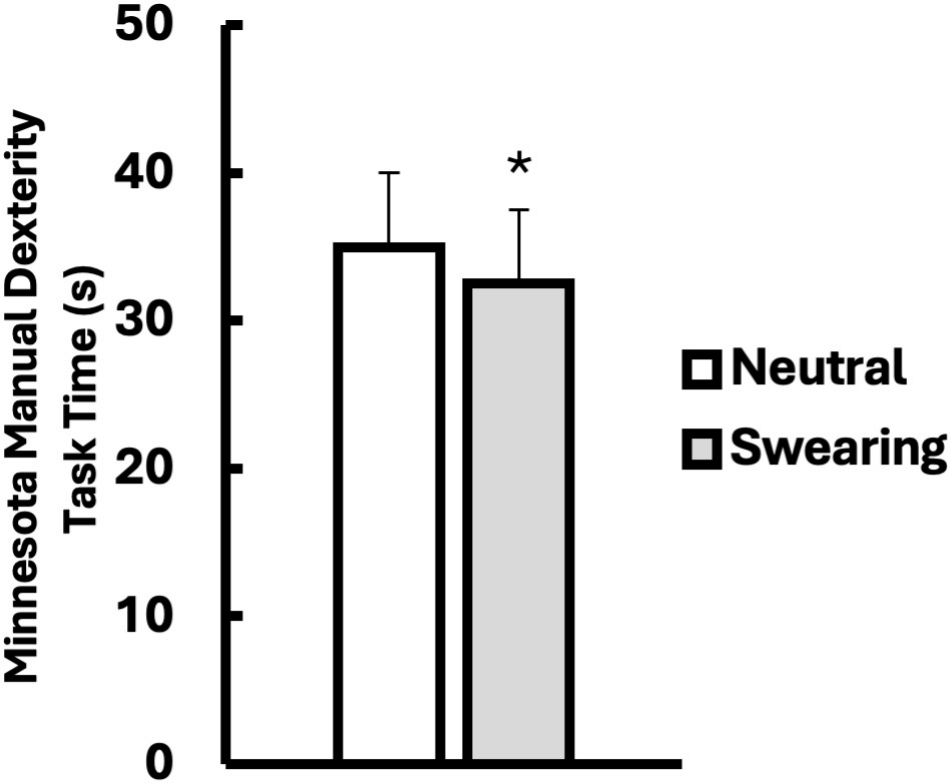
Minnesota Manual Dexterity Task times (s) when repeating a neutral (white) versus a swear word (gray). Data are presented as mean ± SD. *Indicates significantly different from Neutral (*p* < 0.05).

All statistics were analyzed using IBM SPSS Statistics for Windows, Version 22. The criterion for statistical significance was set at an alpha level of *p* < 0.05. Descriptive data are shown in Table 1. A one-way within subjects ANOVA was conducted to compare the effects of swearing on MMDT completion time compared to the neutral word condition. Results indicated a significant difference between the swear word and neutral word conditions, *F*(1, 60) = 22.002, *p* < 0.001, η^2^_*p*_ = 0.268 (Figure 2).

**TABLE 1:** Descriptive data.

Variable	*M*	SD
Age (years)	21.61	4.26
**Minnesota Manual Dexterity Task (seconds)**		
Neutral	34.86	7.02
Swearing	32.52	5.95

*N* = 61.

A one-way within subjects ANOVA was conducted to compare the effects of swearing on reaction times in the SSRT compared to the neutral word condition. There was no significant difference between reaction times in the SSRT between swearing and neutral word conditions, *F*(1, 60) = 1.51, *p* = 0.699, η^2^_*p*_ = 0.003. A one-way within subjects ANOVA was conducted to compare the effects of swearing on SSRT success rates compared to the neutral word condition, finding no significant difference between the success rates in the SSRT between swearing and neutral word conditions, *F*(1, 60) = 0.975, *p* = 0.328, η^2^_*p*_ = 0.016.

### Discussion

Hypothesis (1), that manual dexterity would be improved by repeating a swear word just prior to completing the task, was supported. Faster completion times on the MMDT occurred when participants repeated a swear word, compared to a neutral word. However, hypothesis (2), that swearing would lead to increased state disinhibition, was not supported. Despite faster manual dexterity scores, the SSRT reaction times and success rates, as operational definitions of state disinhibition, did not differ in the swearing and neutral word conditions. This suggests that the beneficial effect of swearing on manual dexterity was not mediated by state disinhibition. However, it is possible that the SSRT task lacks sufficient sensitivity to detect changes in state disinhibition caused by swearing. Previous evidence does suggest swearing increases psychological traits linked to state disinhibition, such as improved self-confidence, mood, and psychological flow (Ballmann et al., 2024; Stephens et al., 2022).

Experiment 1 tested whether the beneficial effects of swearing on manual dexterity could be explained by increased state disinhibition, a proposed mechanisms previously linked to swearing’s effect on physical performance (Beck et al., 2024; Stephens et al., 2022). While swearing improved manual dexterity, no change in state disinhibition was observed, raising questions about the underlying mechanism.

Given the potential of swearing, a cheap, readily available, sustainable, drug-free intervention to confer benefits on human performance, a second experiment was conducted as a conceptual replication, shifting focus from mechanistic-testing to boundary-testing. Specifically, Experiment 2 examined whether swearing’s effect on manual dexterity was moderated by habituation (i.e., daily swearing frequency), a factor known to blunt swearing’s impact on pain tolerance (Stephens and Umland, 2011).

## Experiment 2

A second experiment was conducted to conceptually replicate the findings of Experiment 1 and further investigate the theory that swearing functions as an effective psychological warm-up. Although Experiment 1 included a state disinhibition task, Experiment 2 omitted this component for a couple reasons. First SSRT outcomes in Experiment 1 did not show sensitivity to changes in disinhibition due to swearing. Second, given prior studies showing mixed effects of swearing on disinhibition (Beck et al., 2024; Stephens et al., 2022), a theoretically grounded moderator was prioritized: daily swearing frequency (Stephens and Umland, 2011). Examining a plausible moderating factor of whether swearing-induced improvements in manual dexterity might be reduced by habituation extends the generalizability and theoretical reach of these findings.

The effect size and statistical power estimates from Experiment 1 guided the sample size for Experiment 2. It was hypothesized (1) that manual dexterity scores would improve with swearing compared to repeating a neutral word. Additionally, it was hypothesized (2) that a habituation effect would be observed as an interaction effect, such that the effects of swearing on manual dexterity would depend on daily swearing habits, with infrequent swearers benefiting more than frequent swearers.

### Methods

#### Participants

Forty-two participants (26 females; *M* = 28.31 years, SD = 9.44 years) were recruited from the Samford University and Birmingham, Alabama communities via convenience sampling. Exclusion criteria included individuals less than 18 or older than 99 years old, as well as those with any health condition affecting manual dexterity. The sample size was guided by data from Experiment 1 using the G*Power app (Faul et al., 2009). Informed consent was obtained prior to participation, and the protocol was approved by the Samford University Institutional Review Board (approval number EXPD-HP-24-SUM-1). After providing informed consent, participants recorded their age, gender, dominant hand, and answered the question “How often do you swear in daily life?”.

#### Materials

##### Nine-Hole Peg Test (9-HPT)

**FIGURE 3:**
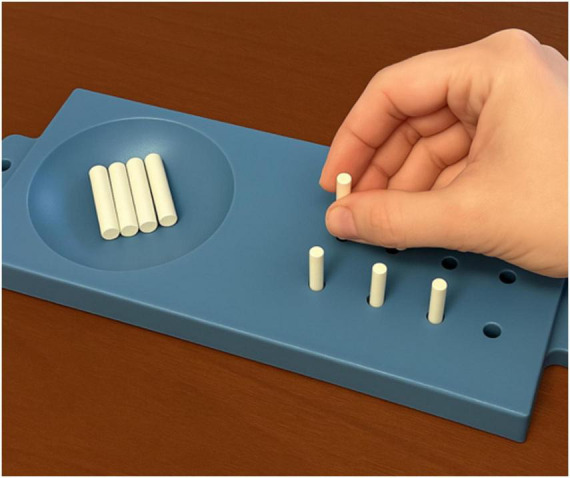
Nine-Hole Peg Test. Figure created by ChatGPT-4.

The Jamar Nine-Hole Peg Test (Jamar Health Products, Greendale, WI) was used to measure manual dexterity (Figure 3). This test is widely used and has demonstrated good to excellent validity and reliability in healthy populations (Grice et al., 2003). Participants were required to use their non-dominant hand to sequentially place and remove all nine pegs, as quickly as possible. The total completion time was recorded in seconds.

##### Vocalization

Participants self-selected their swear and neutral words as described in Experiment 1.

#### Design and procedure

A repeated measures design was used, with the conditions (swear word, neutral word) randomized across participants. The dependent variable was time taken to complete the 9-HPT.

Participants completed two trials of the 9-HPT, one in each condition (swear word, neutral word), with a 5-min rest between trials. Prior to each trial, they repeated their self-selected word for 15 s.

### Results

**FIGURE 4:**
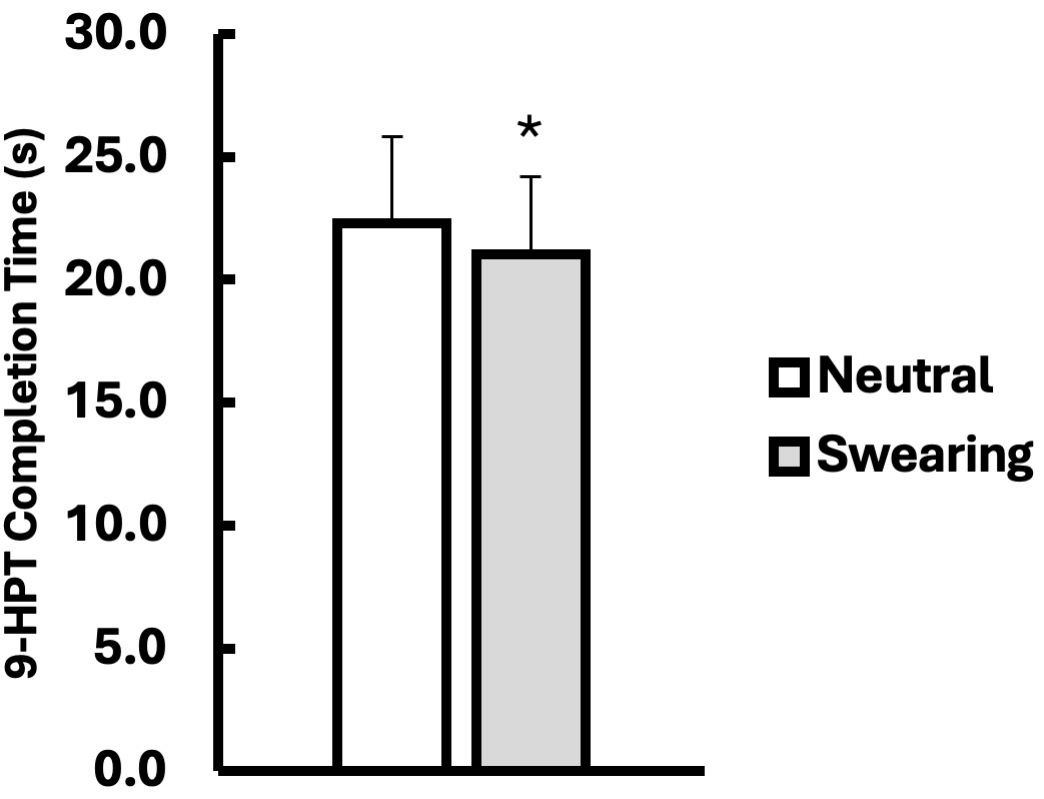
Nine-Hole Peg Test times (s) when repeating a neutral (white) versus a swear word (gray). Data are presented as mean ± SD. *Indicates significantly different from Neutral (*p* < 0.05).

All statistics were analyzed using IBM SPSS Statistics for Windows, Version 22, with statistical significance set at an alpha level of *p* < 0.05. Descriptive data are presented in Table 2. Participant responses to the question “How often do you swear in daily life?” were converted into a continuous daily swearing frequency score (e.g., swearing 6x/day = 6). Ambiguous responses such as “too many times,” “frequently,” or “some” were conservatively assigned a score of 2 (the median value). A repeated-measures general linear model (GLM) was conducted to examine the effects of vocalization (swearing vs. neutral word) and daily swearing frequency scores on 9-HPT times. There was a significant main effect of vocalization, *F*(1, 40) = 4.717, *p* = 0.036, η^2^_*p*_ = 0.105 (Figure 4), supporting the hypothesis that manual dexterity scores would improve with swearing. However, there was a non-significant interaction between vocalization and daily swearing frequency scores, *F*(1, 40) = 1.032, *p* = 0.316, η^2^_*p*_ = 0.025. Additionally, there was no main effect for daily swearing frequency scores, *F*(1, 40) = 2.122, *p* = 0.153.

**TABLE 2:** Descriptive data.

Variable	*M*	SD
Age (years)	28.31	9.44
**9-Hole Peg Test (seconds)**		
Neutral	22.31	3.52
Swearing	21.05	3.15

*N* = 42.

### Discussion

Hypothesis (1), that manual dexterity scores would improve with swearing compared to repeating a neutral word, was supported. Participants completed the 9-HPT faster when repeating a swear word, suggesting that swearing may enhance fine motor performance. However, contrary to hypothesis (2), daily swearing frequency did not moderate this effect. That is, participants who reported swearing more frequently in daily life did not exhibit diminished performance benefits.

## General discussion and conclusion

To our knowledge, these experiments are the first to investigate the effects of swearing on manual dexterity. The primary goal was to determine whether swearing could function as an effective psychological warm-up. Across both experiments, participants demonstrated statistically significant improvements in performance on manual dexterity tasks when repeating a swear word compared to a neutral word. These findings support the theory that swearing may serve as a mental warm-up strategy. In Experiment 1, participants completed the MMDT an average of 6.7% faster in the swearing condition. Similarly, in Experiment 2, participants completed the 9-HPT an average of 5.7% faster in the swearing condition, further reinforcing this effect. Although these findings are novel, they align with previous research showing beneficial effects of swearing on pain tolerance (Stephens et al., 2009) and physical performance (Jiannine and Antonio, 2023; Stephens et al., 2018, 2022). By extending these findings to manual dexterity, this study contributes to the growing body of evidence supporting the positive physiological, social, and psychological effects of swearing (Stapleton et al., 2022).

While these experiments suggest swearing improves manual dexterity, the underlying mechanism behind swearing’s beneficial effects remains unclear. Experiment 1 was designed to test whether state disinhibition mediated the observed improvements. However, despite improved manual dexterity performance on the MMDT, state disinhibition, as measured by the SSRT, did not differ between the swearing and neutral word conditions. Prior research on swearing’s effect on state disinhibition has been mixed, with some studies showing an increase and others showing no effect (Beck et al., 2024; Stephens et al., 2022). One possible explanation is that state disinhibition underlies the observed swearing-induced improvement in manual dexterity, but the research design lacked the sensitivity to detect changes in variables linked to state disinhibition. Swearing as a psychological warm-up to increase state disinhibition via pathways of improved arousal, attentional control, and self-confidence remains a plausible explanation for its observed benefits on manual dexterity. Furthermore, swearing is known to trigger sympathetic activation, the “fight or flight” response characterized by increased heart rate, blood pressure, respiratory rate, pupillary dilation, and skin conductance. For example, Harris et al. (2003) demonstrated that skin conductance is higher when individuals swear compared to repeating neutral words, and swearing also produces greater speech-evoked pupillary responses than vocalizing neutral words (Reilly et al., 2020). Furthermore, Shafto et al. (2024) reported a strong correlation between the tabooness of a word and its self-reported arousal rating. Because physiological arousal is closely linked to task engagement (Behnke et al., 2021), it plausible that this sympathetic activation explains the beneficial effects of swearing on manual dexterity.

Experiment 2 aimed to conceptually replicate the findings in Experiment 1, which demonstrated that swearing had a beneficial effect on manual dexterity, and to expand the scope of this topic by assessing whether the relationship between swearing and manual dexterity is moderated by daily swearing frequency. Experiment 2 supports the results of Experiment 1, showing that swearing improved performance on the 9-HPT. However, the hypothesized moderation effect was not observed: the beneficial effects of swearing on manual dexterity did not depend on daily swearing frequency. The absence of a habituation effect contrasts with previous findings in other domains. For example, Stephens and Umland (2011) reported that frequent swearing reduced the analgesic benefits of swearing during a cold pressor task. Similarly, MacKay et al. (2004) found that repeating exposure to swear words diminishes their emotional arousal.

While this study provides novel insights into the potential that swearing may serve as a psychological warm-up and its impact on manual dexterity tasks, several limitations should be acknowledged. These experiments were not pre-registered and should therefore be considered exploratory, although the conceptual replication in Experiment 2 of the effect shown in Experiment 1 provides some assurance that the effect of swearing on manual dexterity is repeatable. Additionally, although a randomized repeated measure design was employed, consistent with prior research in this field (Hay et al., 2024; Jiannine and Antonio, 2023; Stephens et al., 2022), the relatively small homogenous samples of primarily university students may limit the generalizability of these findings to broader populations and contexts. Importantly, this study did not test individuals with dexterity deficits. Future research should include populations with health conditions known to negatively affect manual dexterity (e.g., stroke, Parkinson’s disease, or rheumatoid arthritis). Future research should aim to replicate these findings in pre-registered studies with larger and more diverse samples to better understand the effects of swearing as a psychological warm-up on manual dexterity. Additionally, the two experiments presented here were conducted in different countries: Experiment 1 in the United Kingdom and Experiment 2 in the United States. While swearing is culturally embedded in both British and American English, variations in word choice, usage frequency, contextual norms, and perceived offensiveness (Beers Fägersten, 2012; McEnery et al., 2023) may influence the psychological and behavioral impact of swearing. Such factors, including participants’ baseline attitudes toward swearing and culturally specific swearing practices, represent potential confounding variables that should be addressed in future research. Incorporating questionnaires about participants’ habitual swearing patterns, preferred swear words, or reasons for swearing in daily life may help account for these cultural nuances. Another important consideration is ecological validity. Both experiments were conducted in controlled laboratory settings, and it remains unclear whether the observed improvements in manual dexterity would translate to real-world environments. Investigating potential mechanisms, including sympathetic activation and psychological factors, should be a priority in future research to elucidate how swearing impacts manual dexterity.

This paper presents two experiments demonstrating that swearing can enhance manual dexterity, supporting the theory that swearing may serve as a psychological warm-up. Participants completed both the Minnesota Manual Dexterity Task and the Nine-Hole Peg Test more quickly after repeating a swear word compared to a neutral word. These findings suggest that swearing may have broader applications beyond its known effects on pain tolerance and physical performance.

## Data Availability

The datasets presented in this study can be found in online repositories. The names of the repository/repositories and accession number(s) can be found below: https://osf.io/4rn62/.
